# Negative correlation between the nuclear size and nuclear Lamina component Lamin A in intraductal papillary mucinous neoplasms of the pancreas

**DOI:** 10.3389/pore.2022.1610684

**Published:** 2022-12-06

**Authors:** Tamaki Hiroe, Shunichi Moriya, Sayaka Kobayashi, Yoshimi Nishijima, Akira Watanabe, Ken Shirabe, Hayato Ikota, Hideaki Yokoo, Masanao Saio

**Affiliations:** ^1^ Laboratory of Histopathology and Cytopathology, Department of Laboratory Sciences, Graduate School of Health Sciences, Gunma University, Maebashi, Gunma, Japan; ^2^ Department of Hepatobiliary and Pancreatic Surgery, Graduate School of Medicine, Gunma University, Maebashi, Gunma, Japan; ^3^ Clinical Department of Pathology, Gunma University Hospital, Maebashi, Gunma, Japan; ^4^ Department of Human Pathology, Graduate School of Medicine, Gunma University, Maebashi, Gunma, Japan

**Keywords:** Emerin, Lamin A, nuclear morphology, intraductal papillary mucinous neoplasm, computer-assisted image analysis

## Abstract

**Background:** The nuclear laminar protein Lamin A and inner nuclear membrane protein Emerin plays important role in sustaining nuclear structure. However, They have not investigated the significance of these proteins for development of pancreatic intraductal papillary mucinous neoplasm (IPMN).

**Methods:** We examined pancreatic IPMN specimens for nuclear morphology and nuclear protein expression pattern of Lamin A and Emerin. Forty-two IPMN specimens were included, with 30 classified as intraductal papillary mucinous adenoma (IPMA) and 12 as intraductal papillary mucinous carcinoma (IPMC).

**Results:** Classification according to histological subtype revealed that 26 specimens were of the gastric subtype (1 IPMC case), 8 were pancreatobiliary (6 IPMC cases), 6 were intestinal (3 IPMC cases), and 2 were oncocytic (all cases were IPMC). The frequency of IPMN subtypes in this study seemed to agree with those in previous reports. We analyzed Feulgen staining sections for nuclear morphological analysis using computer-assisted image analysis. Nuclear area and perimeter were significantly larger in IPMC than in IPMA. Finally, we examined the positive ratios of Lamin A and Emerin in immunohistochemical staining sections by image analysis. We found a negative correlation between the nuclear size and Lamin A-positive ratio, which was significantly lower in IPMC than that in IPMA. However, no significant correlation was observed between nuclear size and Emerin expression was observed, and no differences were found in the Emerin-positive ratio between IPMA and IPMC.

**Conclusion:** Our results suggest that a decreased Lamin A positive ratio induces nuclear enlargement in adenomas, which thereby induce promotion to carcinomas. Furthermore, Lamin A expression can be a reliable biomarker for distinguishing between IPMC and IPMA.

## Introduction

Pancreatic intraductal papillary mucinous neoplasm (IPMN) is a type of pancreatic duct epithelial tumor that dilates the pancreatic duct by mucus retention [1,2]. IPMN is classified into two types based on the type of malignancy: intraductal papillary mucinous adenoma (IPMA) and intraductal papillary mucinous carcinoma (IPMC) [3]. IPMN is classified into four subtypes (gastric, intestinal, pancreatobiliary, and oncocytic) based on the morphological features and immunohistochemical characteristics of mucin glycoprotein [2,4,5]. In detail, gastric-type IPMN is characterized as MUC1(−), MUC2(−), MUC5AC(−), and MUC6(+); intestinal type as MUC1(−), MUC2(+), MUC5AC(+), and MUC6(+); and pancreatobiliary type as MUC1(+), MUC2(-), MUC5AC(+), and MUC6(+). Oncocytic type-IPMN has an eosinophilic cytoplasm and MUC1(+), MUC2(−), MUC5AC(+), and MUC6(+) phenotype. IPMN subtype and tumor malignancy have recently been found to be closely related. Among the IPMN subtypes, the gastric type tends to have good prognosis, whereas the pancreatobiliary type tends to have a poorer one [6].

Lamin and Emerin are protein components of the nuclear envelope. Lamin is one of the intermediate filaments that make up the nuclear lamina. Lamin is divided into two types: Lamin A/C and Lamin B. Lamins A and C are encoded by the same gene, but their splicing positions are different. That is, Lamins A and C have common N-terminal 566 amino acids, but Lamin C is a truncated form of Lamin A [7]. Thus, Lamins A and C are sometimes termed Lamin A/C and evaluated as one. Lamin A/C has been found to interact with Emerin [8,9]. Emerin has a single transmembrane domain passing through the inner nuclear membrane and interacts with other nuclear envelope proteins. Lamin A and Emerin are involved in nuclear stiffness. For example, the decreased expression of Lamin A and/or Emerin results in nuclear atypia and increases the appearance of nuclear bleb-positive cells [10,11]. Lamin A-deficient mouse embryo fibroblasts have shown significantly high nuclear deformation under strained conditions [11]. Nuclear plasticity may be involved in cancer cell migration into tissue mesenchyme. A study using human ovarian cancer cell lines indicated that decreased Lamin A expression promoted migration of the tumor cell, whereas Lamin A overexpression suppressed it [12]. Several recent studies suggest that Lamin and Emerin expression is involved in tumor malignancy [13]. Decreased Lamin A/C and Emerin expression tends to be correlated with tumor grade in osteosarcoma cell lines.

In addition, a negative correlation has been established between decreased Lamin A/C expression and survival in osteosarcoma and breast cancer [13,14,15]. Additionally, a negative correlation has also been found between decreased Lamin A expression and patient survival in ovarian cancer and colorectal cancer [12,16,17]. By contrast, Lamin B1 overexpression results in a survival ratio of less than 30 months in colorectal cancer patients [18]. Of the Lamins, Lamin A seems to be the most closely related to tumor malignancy.

However, no reports have described the relationship between IPMN and the alteration of nuclear membrane proteins such as Lamins and Emerin. Thus, in the present study, we analyzed how Lamin A and Emerin contribute to tumor progression in human IPMN cases.

## Materials and methods

### Cases

We selected specimens from 42 IPMN cases that were surgically resected at Gunma University Hospital (Maebashi, Gunma, Japan) between January 2005 and December 2018. The formalin-fixed paraffin-embedded (FFPE) tissue block used for pathological diagnosis in daily practice was used in this study.

### Ethical approval

The present study was approved by the Gunma University Ethical Review Board for Medical Research Involving Human Subjects of Gunma University School of Medicine (GUERB) (Approval No. HS2019-041), and the written notification for the current study was presented publicly on the webpage of Gunma University Hospital. Furthermore, the possibility to decline participation in this study was provided according to the Ethical Guidelines for Medical and Health Research Involving Human Subjects of the Japanese government (Ministry of Education, Culture, Sports, Science and Technology and Ministry of Health, Labour and Welfare)

### Specimen preparation

Four paraffin sections 4 μm thick were prepared with a sliding microtome (REM-710; Yamato Kohki Industrial, Saitama, Japan). The paraffin blocks were chilled with a Paracooler (PC-110; REM-710; Yamato Kohki Industrial) during slicing.

### Hematoxylin–eosin staining

Sections were deparaffinized with xylene three times for 5 min each; then rehydrated with 100% ethanol for 1 min, 95% ethanol for 1 min, and 70% ethanol for 1 min; and rinsed in running water for 1 min. After staining with Mayer’s Hematoxylin Solution (New Hematoxylin Type M; Muto Pure Chemicals, Tokyo, Japan) for 10 min, sections were rinsed in running water for 1 min. Subsequently, sections were stained with eosin solution (New Eosin Type M; Muto Pure Chemicals) and rinsed in running water for 5 s. Dehydration (70% ethanol for 1 min, 95% ethanol for 1 min, and 100% ethanol for 1 min) and permeabilization (xylene three times for 5 min each time) were performed. Sections were sealed with hydrophobic mounting medium (New Eosin Type M; Muto Pure Chemicals) before observation.

### Feulgen reaction

Sections were de-paraffinized with xylene three times for 5 min each time, rehydrated with ethanol (100% for 1 min, 95% for 1 min, and 70% for 1 min), rinsed in running water for 1 min, and rinsed with distilled water for 1 min. Afterward, sections were soaked in 5 M hydrochloric acid at 30°C for 40 min to remove the purine bases of DNA and then rinsed two times with 1 ml Schiff’s reagent (Cold Schiff’s Reagent; Cat. No. 40932, Muto Pure Chemicals) at room temperature (R/T). A further 1.5 ml Schiff’s reagent was added to the sections and set aside for 60 min. After the reagent was tapped off, sections were treated thrice with 1 ml sulfurous acid solution (Muto Pure Chemicals) for 2 min each time. After rinsing in running water, dehydration (70% ethanol for 1 min, 95% ethanol for 1 min, and 100% ethanol for 1 min) and permeabilization (xylene three times for 5 min each) were performed. Sections were sealed with hydrophobic mounting medium before use.

### Immunohistochemistry

Sections were deparaffinized with xylene three times for 5 min each, rehydrated (100% ethanol for 1 min, 95% ethanol for 1 min, and 70% ethanol for 1 min), and rinsed in running water and with distilled water for 1 min each.

To retrieve antigens, sections were set in a heat-resistant rack placed inside a heat-resistant container and immersed in 1,500 ml of antigen retrieval reagent (1:200 dilution; Immunosaver; Nissin EM, Tokyo, Japan). The container was placed in an electric hot pot (PDR-G221; Tiger, Osaka, Japan) and heated to 98°C. The temperature was kept at 98°C for 40 min using the warming function of the hot pot. The container was allowed to remain inside the pot for a further 30 min in the residual heat. From the next step onward, we used automatic immunostaining equipment (HISTOSTAINER; Nichirei Biosciences, Tokyo, Japan). In detail, the specimens were treated for 20 min at R/T with 200 ml of 2% normal goat serum (ab7481, Abcam, Cambridge, United Kingdom) in phosphate-buffered saline (PBS) to block non-specific reaction. After blowing out the serum on the slides with an air blow of the staining agent, 200 ml primary antibodies (Table 1) were coated onto the slides for 1 h at R/T. After rinsing in PBS, 200 μl secondary antibody (Histofine Simple Stain MAX-PO [M] for HISTOSTAINER; Code 724132, Nichirei Biosciences) were coated onto slides for 30 min at R/T. After rinsing in PBS, color was developed twice with 3.3′-diaminobenzidine (DAB) substrate (Histostain DAB substrate in kits for HISTOSTAINER; Code 725191, Nichirei Biosciences) for 5 min each time. After rinsing in DW, sections were counterstained with Mayer’s Hematoxylin Solution (Histostain Mayer’s Hematoxylin Solution for HISTOSTAINER; Code 715081, Nichirei Biosciences). After removing the sections from the equipment, dehydration and permeabilization were performed. Finally, sections were sealed with hydrophobic mounting medium.

**TABLE 1:** Primary antibodies used.

Antigen	Clone	Species	Concentration/dilution	Catalog No., company, location
Lamin A	133A2	Mouse monoclonal	2 μg/ml	ab8980, Abcam, Cambridge, United Kingdom
Emerin	CL0201	Mouse monoclonal	2 μg/ml	NBP2-52876, Novus Biologicals, Centennial, CO, US
MUC1	HMFG1	Mouse monoclonal	2 μg/ml	ab70475, Abcam
	(aka 1.10.F3)			
MUC2	SPM512	Mouse monoclonal	1:800	ab231427, Abcam
MUC5AC	45M1	Mouse monoclonal	2 μg/ml	ab3649, Abcam
MUC6	MUC6/916	Mouse monoclonal	2 μg/ml	ab212648, Abcam

### Whole-slide imaging

Whole slide imaging (WSI) of entire sections was performed using a virtual slide scanner (Nano Zoomer-SQ, C13140-01, Hamamatsu Photonics K.K., Shizuoka, Japan). The settings for image capture were as follows: Object lens: 20× N.A. 0.75; scan mode: 40× mode; maximum capture size: 26 × 76 mm; pixel: 0.23 µm/pixel; light force: light-emitting diode; and image storage format: manual focus mode. We then randomly selected five fields of 40× WSI images as TIFF files using image-viewing software (NDP.view2; U12388-01, Hamamatsu Photonics K.K., Shizuoka, Japan).

### Image analysis

For images of Feulgen staining specimens, TIFF files were converted to MRXS files using image converter software (version 1.14 3DHISTECK Ltd., Budapest, Hungary). Quant Center HistoQuant module version 1.15.4 (3DHISTECK Ltd.) in Pannoramic Viewer (3DHISTECK Ltd.) was then used for image analysis. The conditions for analysis were as follows: Noise reduction, Gauss: 1; hue: 274–322; saturation: 3–24; separation: 10; fill holes: Yes; size: 50–499; and shape: 0–1. We ensured that the computer identified every detection area manually on the monitor. Individually detected nuclei were selected, and only their data were used for analysis. All other areas, including areas with more than one nucleus and non-nuclear areas, were excluded from the analysis.

For the detection of Lamin A and Emerin, TIFF files were analyzed using e-Nucmem version 14 (e-Path, Kanagawa, Japan). The conditions for analysis were as follows: For Lamin A, unit: 0.22 μm/pixel; diameter: 40×; level of positive intensity: 5.0; level of negative intensity: 0.0; minimum area: 14.52 μm^2^; membrane density of positive nuclear membrane: 162.0. In the case of Emerin, unit: 0.22 mm/pixel; diameter: 40×; level of positive intensity: 5.0; level of negative intensity: 4.0; minimum area: 14.52 μm^2^; membrane density of positive nuclear membrane: 182.0.

### Evaluation of staining by pathologists

To evaluate the nuclear size, Lamin A expression, and Emerin expression, two pathologists (MS and HI) manually and independently evaluated the specimens. For nuclear size evaluation, we compared the size of nuclei in IPMN for one of the normal pancreatic ducts. For Lamin A and Emerin expressions, we classified the expression into three levels: marked expression, expressed based on the normal pancreatic duct, and partial loss of expression.

### Statistical analysis

JMP Pro version 12.2.0 (SAS Japan, Tokyo, Japan; https://www.jmp.com/en_us/home.html) was used for statistical analysis. For the correlation analysis, a coefficient of correlation (R) > 0.2 was considered no correlation, 0.2 ≤ R ≤ 0.399 was considered slight correlation, 0.4 ≤ R ≤ 0.699 was considered moderate correlation, and R > 0.7 was considered strong correlation [19].

For multiple pairwise comparisons using a non-parametric test, the Wilcoxon test was used to compare each group, and the chi-square test was used to calculate *p* values. Welch’s t-test was used to compare the averages between two groups. For contingency table analysis, Fisher’s exact test was used to determine *p* values. For β error (acceptance error), we set α = 0.05. Results with *p* values of <0.05 were considered statistically significant.

## Results

### IPMN classification

**FIGURE 1:**
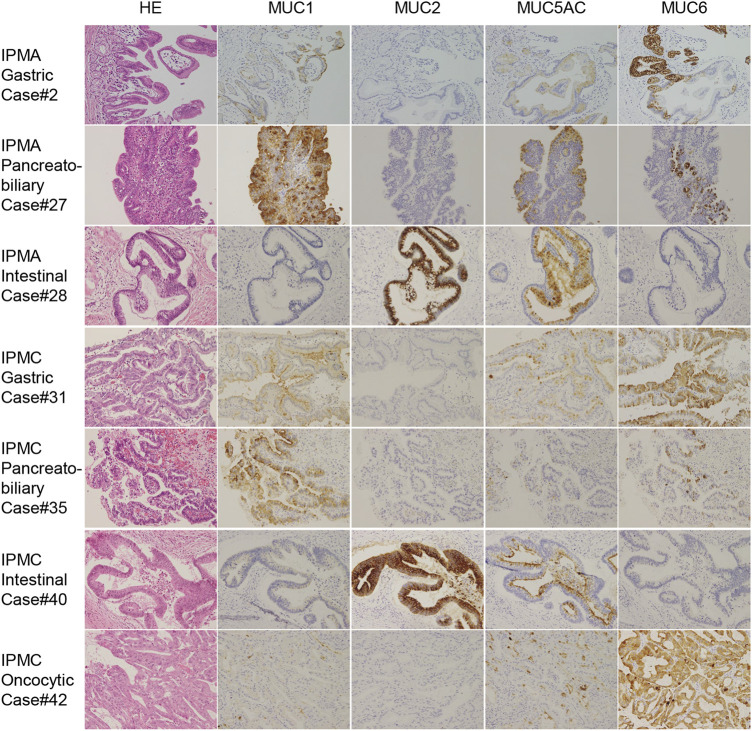
Representative immunohistochemical staining for MUC 1, MUC2, MUC5AC, and MUC6 in IPMA and IPMC subtypes. Because we did not have oncocytic-type IPMA, IPMA of gastric type, pancreatobiliary type, intestinal type, IPMC of gastric type, pancreatobiliary type, intestinal type, and oncocytic type is shown (original magnicfication: ×40).

The clinicopathological features and summary of immunohistochemical staining of tumor specimens collected from 42 patients are shown in Table 2. IPMN specimens were classified as per the histological subtype based on morphological and immunohistochemical characteristics described by the World Health Organization classification [20]. The histological subtypes identified in this study are summarized in Supplementary Table S1. Thirty specimens were classified as IPMA and 12 as IPMC. Twenty-six specimens were classified as gastric type (1 of them was IPMC), 8 pancreatobiliary type (6 were IPMC), 6 intestinal types (3 were IPMC), and oncocytic type (all were IPMC). Representative HE and immunohistochemical staining of each IPMA and IPMC subtype are shown in Figure 1.

**TABLE 2:** Clinicopathological characteristics of tumors in this study.

Subject	Subtype	Gender	Age	T	N	M	IPMA/IPMC	MUC1	MUC2	MUC5AC	MUC6
1	Gastric	F	46				IPMA	−	−	(+)	+
2	Gastric	M	54				IPMA	+	−	+	+
3	Gastric	M	69				IPMA	+/−	−	(+)	+
4	Gastric	M	83				IPMA	+	−	(+)	+
5	Gastric	F	59				IPMA	+	−	(+)	+
6	Gastric	F	70				IPMA	+	−	(+)	+
7	Gastric	M	69				IPMA	+	−	(+)	+
8	Gastric	M	71				IPMA	+	−	+	+
9	Gastric	F	74				IPMA	+	−	+	+
10	Gastric	F	75				IPMA	+	−	+	+
11	Gastric	F	52				IPMA	−	−	(+)	+
12	Gastric	M	75				IPMA	−	−	+	+
13	Gastric	M	68				IPMA	+	−	(+)	+
14	Gastric	M	79				IPMA	−	−	+	+
15	Gastric	M	62				IPMA	+	−	(+)	+
16	Gastric	M	68				IPMA	+	−	+	+
17	Gastric	F	72				IPMA	+	−	+	+
18	Gastric	F	56				IPMA	−	(+)	(+)	+
19	Gastric	M	77				IPMA	+	−	(+)	+
20	Gastric	M	65				IPMA	+	−	(+)	+
21	Gastric	F	71				IPMA	+	−	+	+
22	Gastric	M	71				IPMA	+/−	−	+	+
23	Gastric	M	73				IPMA	+	−	(+)	+
24	Gastric	F	83				IPMA	+	−	(+)	−
25	Gastric	F	74				IPMA	+	−	+	+
26	Pancreatobiliary	M	69				IPMA	+	−	+	(+)
27	Pancreatobiliary	F	62				IPMA	+	−	+	+
28	Intestinal	F	74				IPMA	−	+	+	−
29	Intestinal	M	70				IPMA	−	+	+	(+)
30	Intestinal	M	31				IPMA	−	+	+	(+)
31	Gastric	M	74	Tis	0	0	IPMC	+	−	+	+
32	Pancreatobiliary	F	68	Tis	0	0	IPMC	+	−	(+)	+
33	Pancreatobiliary	M	79	T1	0	0	IPMC	−	−	(+)	−
34	Pancreatobiliary	M	70	T3	0	0	IPMC	+	−	+	+
35	Pancreatobiliary	M	69	T1	0	0	IPMC	+	−	(+)	(+)
36	Pancreatobiliary	M	76	T2	0	0	IPMC	+	−	(+)	+
37	Pancreatobiliary	F	74	T2	1	0	IPMC	(+)	−	(+)	−
38	Intestinal	M	66	T2	0	0	IPMC	+/−	+	+	(+)
39	Intestinal	F	62	Tis	0	0	IPMC	−	+	(+)	(+)
40	Intestinal	M	84	T2	0	0	IPMC	−	+	+	−
41	Oncocytic	F	71	Tis	0	0	IPMC	+	−	+	+
42	Oncocytic	M	78	T2	0	0	IPMC	+	−	(+)	+

M, male; F, female, +/−; faint staining, (+); Partially expressed.

### The majority of gastric-type IPMNs were IPMA, whereas other subtypes were mostly IPMC

Out of 26 gastric-subtype IPMN specimens, 25 were classified as IPMA and only 1 specimen (3.85%) as IPMC. Conversely, for the other subtypes, at least 50% were IPMC: 6 out of 8 specimens (75.0%) in the pancreatobiliary type, 3 out of 6 (50.0%) in the intestinal type, and all 2 specimens (100.0%) in the oncocytic type (Supplementary Table S1). Thus, we subcategorized the subtypes into gastric and nongastric subtypes and examined their differences for the component ratio of IPMA and IPMC using a two-sided Fisher’s exact test. As shown in Supplementary Table S2, a significant difference was found between the gastric and nongastric types (*p* < 0.0001).

### The nuclear area and perimeter in IPMC were significantly larger than those in IPMA both in the total IPMN and nongastric-type IPMN analyses

**FIGURE 2:**
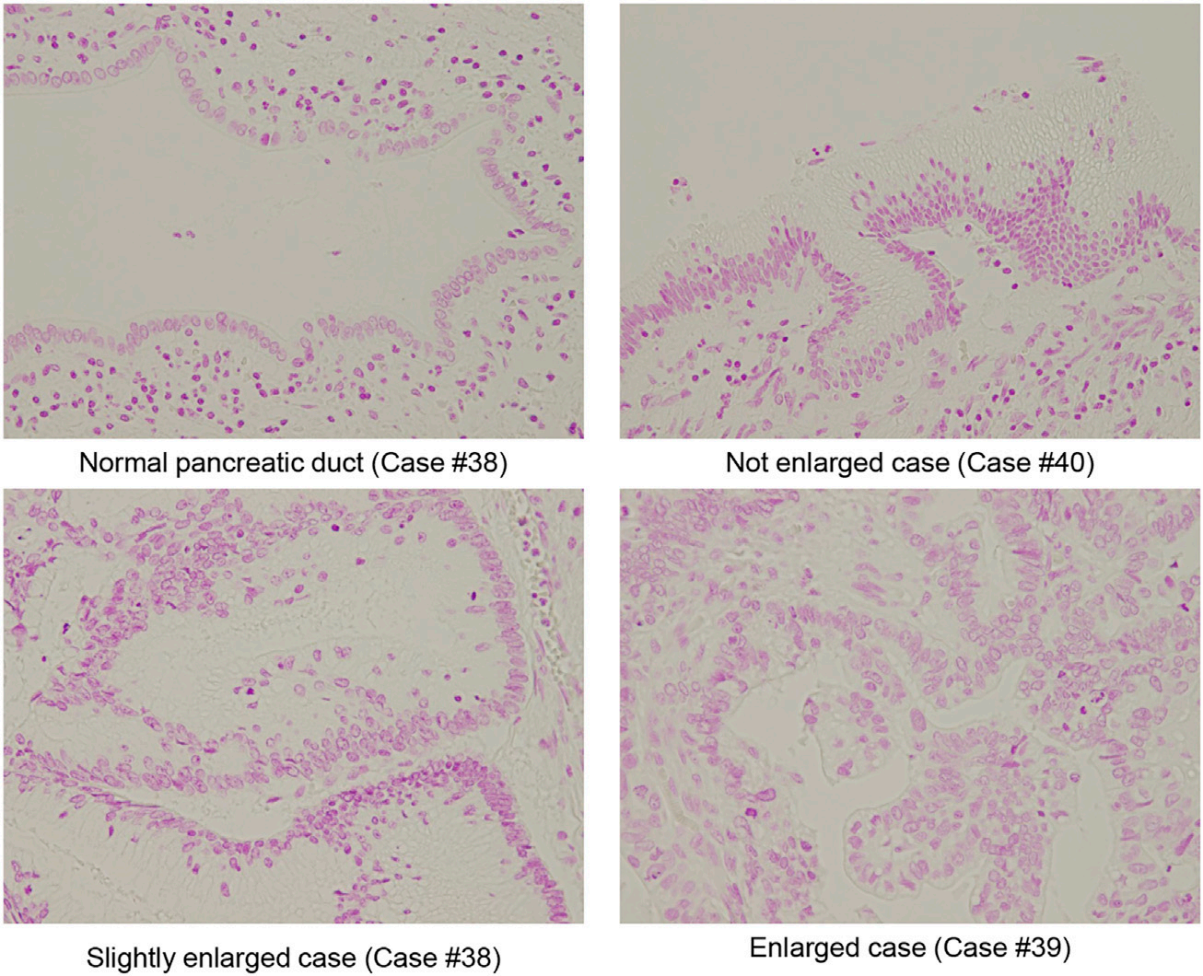
Representative Fuelgen staining of IPMN. Representative size of the normal pancreatic duct, nuclear size with not enlarged IPMA case, nuclear size slightly enlarged IMPA case, and nuclear size with enlarged IPMC case are shown (original magnification: ×40).

**FIGURE 3:**
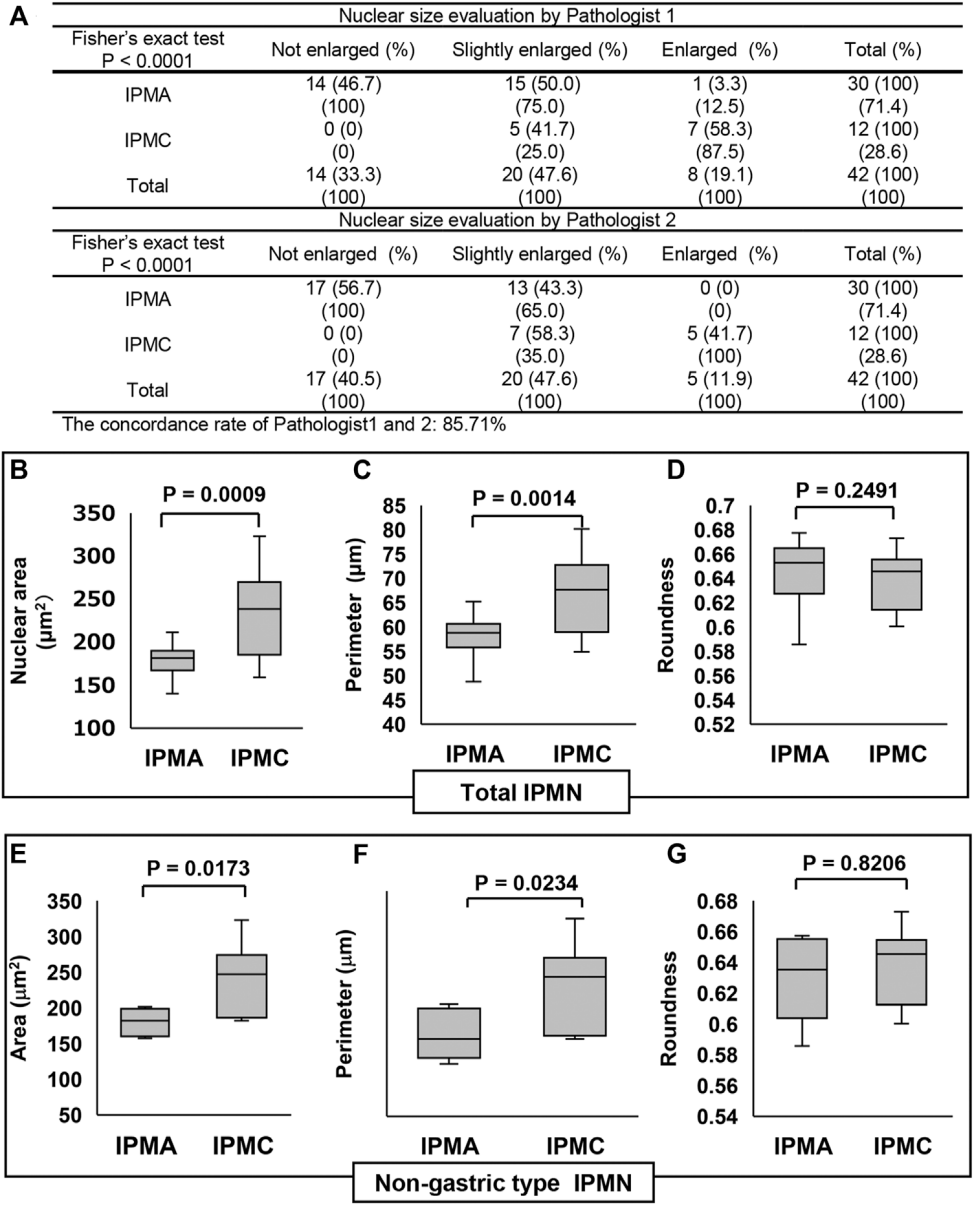
Comparison of the nuclear size in IPMN through manual evaluation and CAIA. **(A)** Contingency table of the nuclear size evaluation by two pathologists between IPMA and IPMC. **(B–D)** Comparisons of the nuclear size, perimeter, and roundness between IPMA and IPMC. **(E–G)** Comparisons of the nuclear size, perimeter, and roundness between nongastric-type IPMA and IPMC **(B, E)** Box plots of nuclear area, **(C, F)** box plots of nuclear perimeter, and **(D, G)** box plots of nuclear roundness. The box with a horizontal line indicates the median and interquartile range. Error bars indicate maximum and minimum values. The mean was evaluated using Welch’s test, and *p* values were calculated using χ^2^ test.

We examined the size and shape of nuclei of the IPMN to determine any differences between IPMA and IPMC not by both manual evaluations of pathologists and CAIA. Representative nuclear findings by Feulgen staining for normal pancreatic duct, IPMA and IPMC, are shown in Figure 2. By manually evaluating Feulgen specimens, a significant difference was observed between IPMA and IPMC for nuclear size by an independent evaluation of two pathologists with an 85.71% concordance rate (Figure 3A). For CAIA, initially, any Feulgen reaction-positive area was automatically measured by CAIA. Thus, areas with a single nucleus were selected manually, and only data for these nuclei were included in statistical analysis. Areas with multiple nuclei (Change Figure 1A to Supplementary Figure S1A) or areas without nuclei (Change Figure 1B to Supplementary Figure S1B) were excluded from further analysis; only areas with a single nucleus (Change Figure 1C to Supplementary Figure S1C) were analyzed. The nuclear parameters were the area, perimeter, and roundness. The mean ± standard deviation (SD) nuclear area of IPMA was 178.06 ± 21.70 μm^2^ and that of IPMC was 235.86 ± 51.16 μm^2^ (Figure 3B), indicating that IPMC has a significantly larger nuclear area than IPMA (*p* = 0.0009). The mean ± SD nuclear perimeter of IPMA was 58.18 ± 4.17 μm, whereas that of IPMC was 67.14 ± 8.15 μm (Figure 3C), indicating that the nuclear perimeter of IPMC was significantly larger than that of IPMA (*p* = 0.0014). Conversely, no differences were observed in nuclear roundness between IPMA and IPMC (0.65 ± 0.022 μm and 0.64 ± 0.025 μm, respectively) (Figure 3D). These data indicated that the nuclear size was significantly changed (enlarged) in IPMC in comparison to IPMA through manual evaluation and CAIA.

The subgroup analysis for nongastric-type IPMN revealed that the mean ± SD nuclear area in nongastric IPMA was 180.01 ± 19.53 μm^2^, whereas that in nongastric IPMC was 242.83 ± 47.30 μm^2^ (Figure 3E), reflecting earlier findings that the mean nuclear area in the latter was significantly larger than that in former (*p* = 0.0173). Similarly, the mean ± SD nuclear perimeter in nongastric IPMA was 59.55 ± 4.51 μm, whereas that in nongastric IPMC was 68.26 ± 7.54 μm (Figure 3F), indicating that the mean nuclear perimeter in the latter was significantly larger than that in the former (*p* = 0.0234). As with earlier findings, no significant difference was observed in nuclear roundness between the nongastric-subtype IPMA and IPMC (0.63 ± 0.029 μm and 0.64 ± 0.025 μm, respectively) (*p* = 0.8206) (Figure 3G).

These data indicated that not only in the total IPMN analysis but also in nongastric-type IPMN analysis that nuclear size was significantly enlarged in IPMC.

### The positive ratio of Lamin A in IPMC was significantly lower than that in IPMA but one of Emerin was not

**FIGURE 4:**
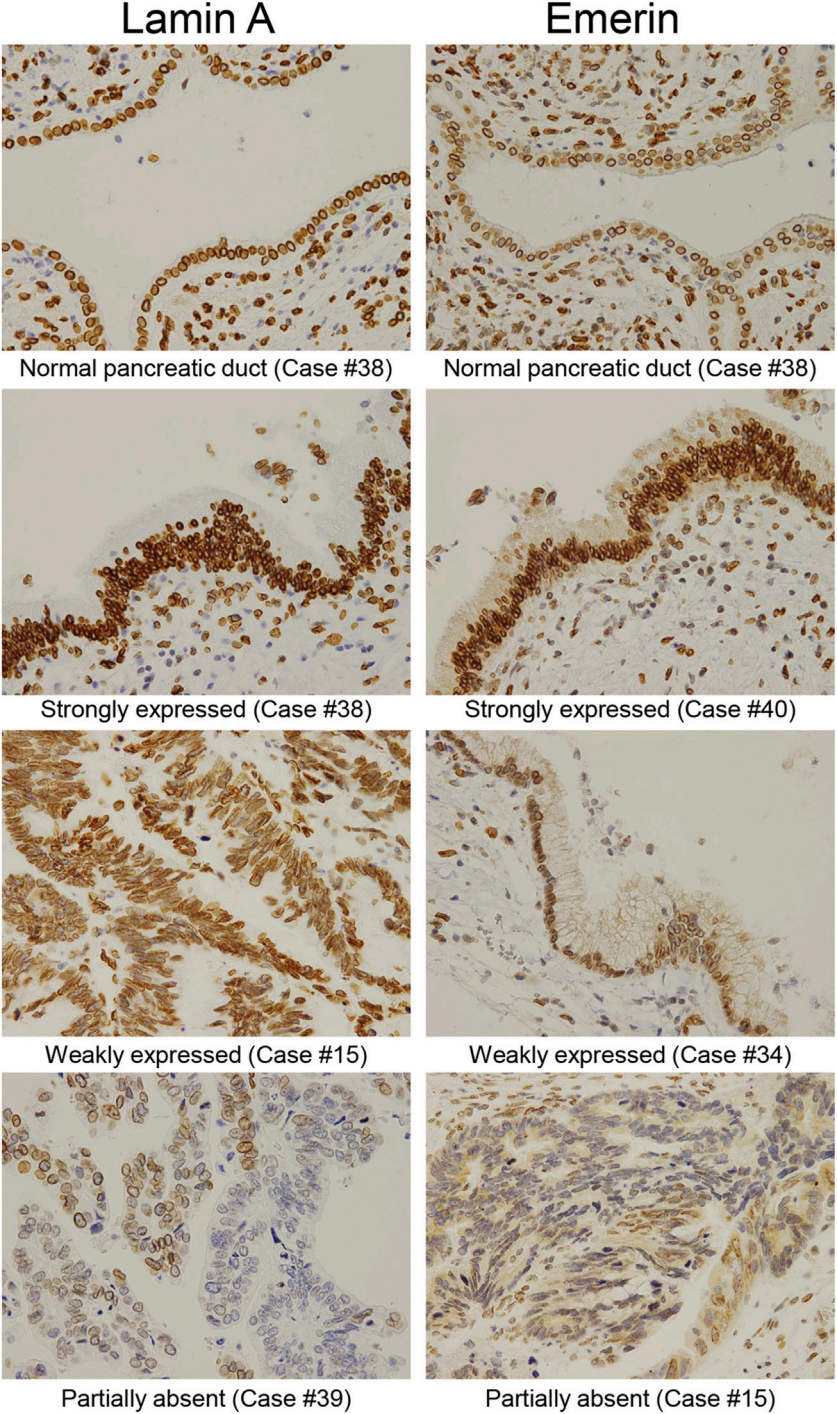
Representative immunohistochemical staining of Lamin A and Emerin in different IPMN expression levels. Representative expression patterns of the normal pancreatic duct in the strongly, weakly, and partly expressed nuclear staining abolished cases were shown for Lamin A and Emerin (original magnification: ×40).

**FIGURE 5:**
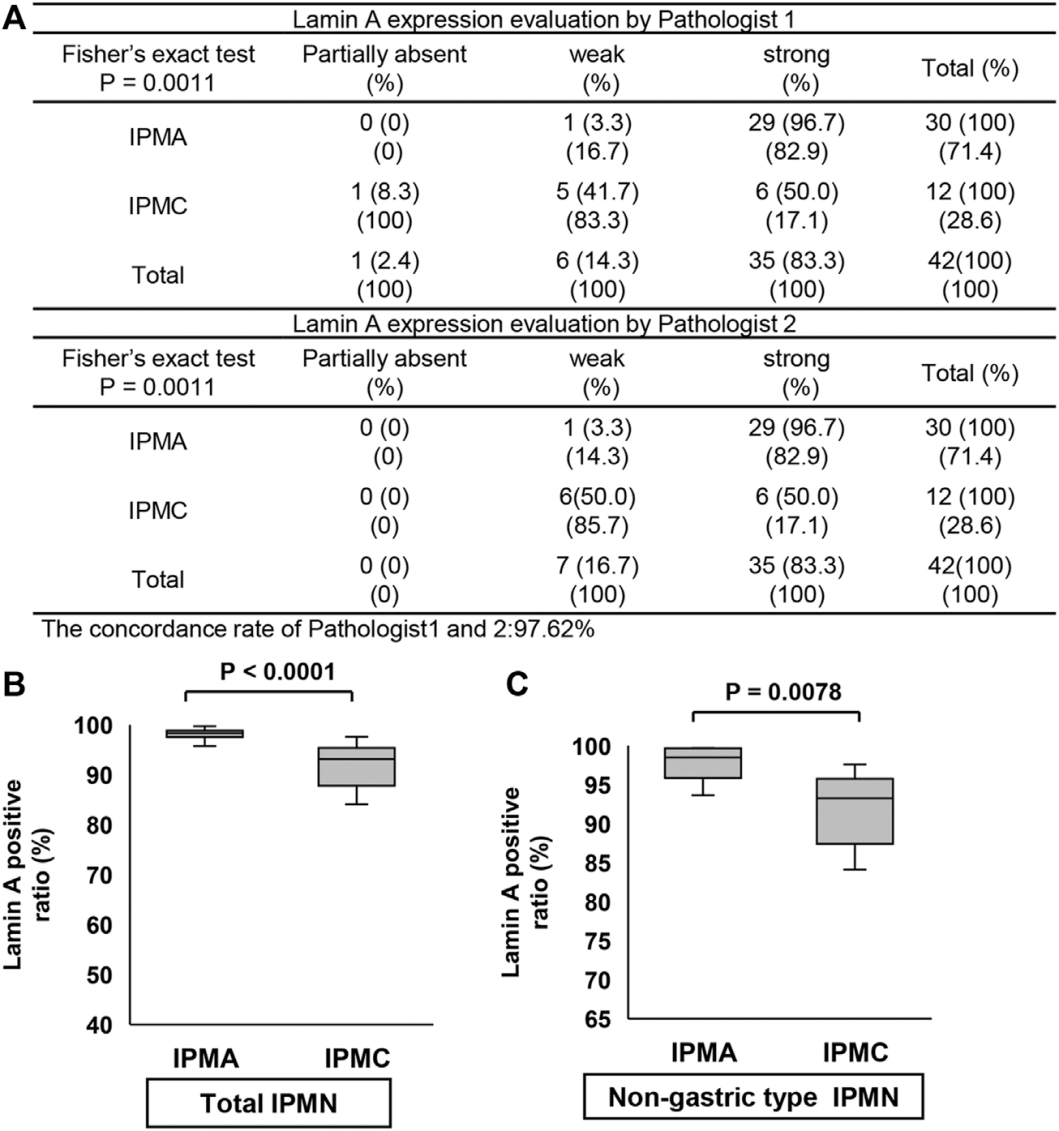
Comparison of Lamin A expression in IPMN through manual evaluation and CAIA. **(A)** Contingency table of Lamin A expression level evaluation by two pathologists between IPMA and IPMC. **(B,C)** Comparison of Lamin A expression between IPMA and IPMC. **(B)** Box plots of Lamin A expression in the total IPMA (left) and IPMC (right). **(C)** Box plots of Lamin A expression in the nongastric-type IPMA (left) and IPMC (right). The horizontal line within the boxes indicates the median and interquartile ranges. Error bars indicate maximum and minimum values. The mean was evaluated using Welch’s test, and *p* values were calculated using χ^2^ test.

We examined the differences in positive ratios of Lamin A and Emerin between IPMA and IPMC through manual evaluation by two pathologists and CAIA. Immunostained Lamin A and Emerin sections were prepared, and their representative staining is shown in Figure 4. A manual evaluation was performed independently by two pathologists. We observed a significant difference between IPMA and IPMC for expression levels of Lamin A (*p* < 0.0001) with a 97.62% concordance rate between the two pathologists (Figure 5A). However, by CAIA, the mean ± SD positive ratio of Lamin A in IPMA was 97.79% ± 2.17%, whereas that in IPMC was 90.02% ± 8.96%, with statistically significant differences (*p* < 0.0001) (Figure 5B). In contrast, no significant difference was observed between IPMA and IPMC for the expression level of Emerin through independent manual evaluation of two pathologists (*p* = 0.0892 and *p* = 0.3695) although the concordance rate was 90.48% (Supplementary Figure S2A). Moreover, no differences in the positive ratio of Emerin were found between IPMA (95.17% ± 5.69%) and IPMC (86.85% ± 19.80%) (*p* = 0.4951) (Supplementary Figure S2B). These data suggest that Lamin A expression in IPMC was significantly lower than that in IPMA both by manual evaluation and CAIA.

Because only one gastric-subtype IPMC specimen was obtained, we could not compare the differences in Lamin A- or Emerin-positive ratio between gastric-subtype IPMA and IPMC. Thus, we prepared a scatter diagram of Lamin A- and Emerin-positive ratios for four categories (gastric-type IPMA, gastric-type IPMC, nongastric-type IPMA, and nongastric-type IPMC) (Supplementary Figure S3). The diagram indicates that Lamin A seemed to be lower in IPMC specimens.

Thus, we examined the positive ratios of Lamin A and Emerin to compare the differences between nongastric-subtype IPMA and IPMC. The Lamin A-positive ratio in IPMA was 97.92% ± 2.48%, whereas that in IPMC was 89.75% ± 9.35%, which showed a statistically significant difference between them (*p* = 0.0078) (Figure 5C). Conversely, no differences were observed in the positive ratio of Emerin between IPMA (89.62% ± 10.29%) and IPMC (89.59% ± 18.22%) (*p* = 0.4964) (Supplementary Figure S2C). These findings suggest that canceration causes nuclear enlargement and a significant decrease in Lamin A-positive ratio in nongastric-subtype IPMC. These data showed manual evaluation findings.

### Lamin A expression level but not Emerin expression level was negatively correlated with nuclear size

**FIGURE 6:**
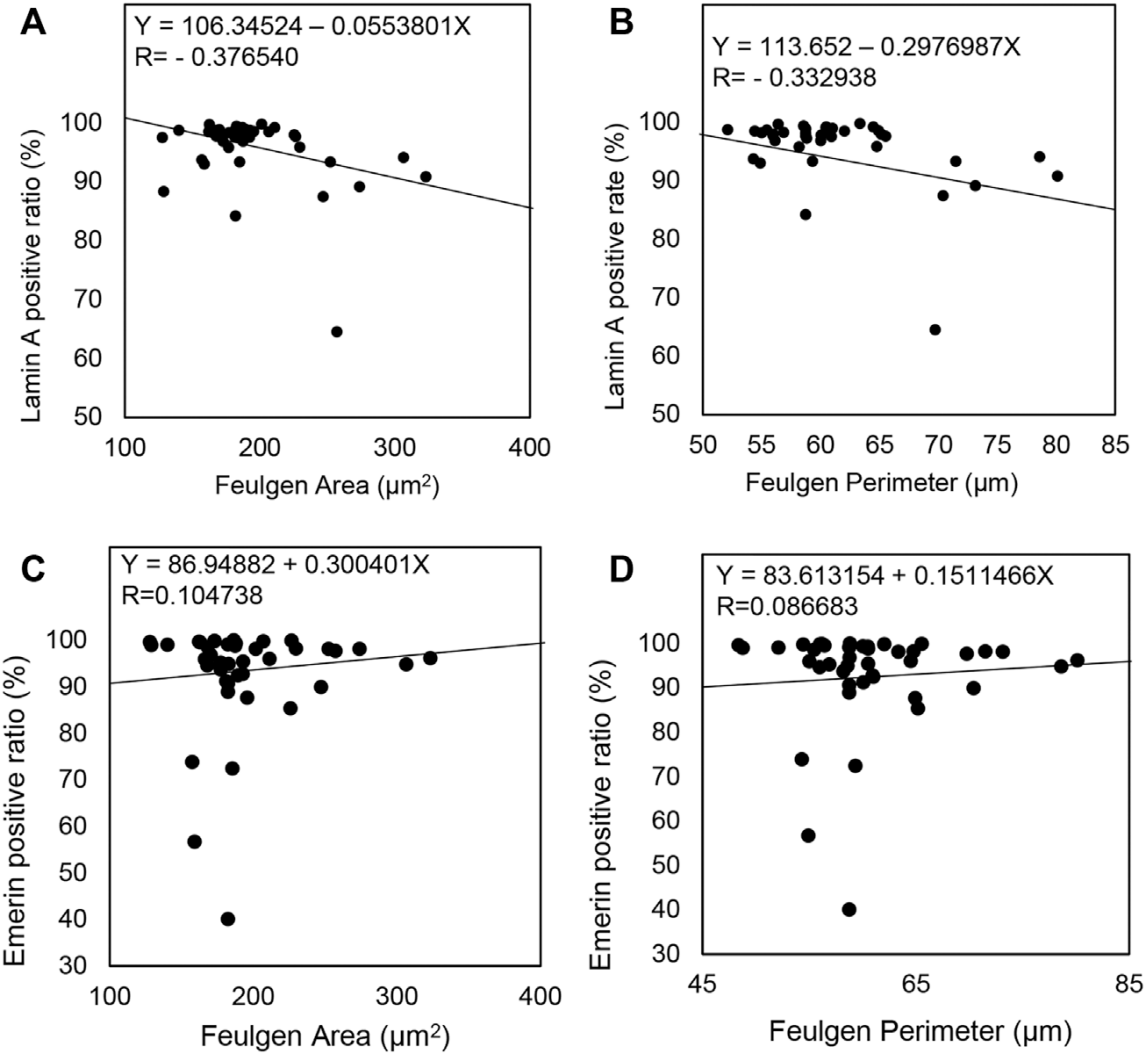
Comparison of Lamin A and Emerin expression with nuclear morphological parameters in all IPMN cases. **(A–D)** A straight line in each scatterplot indicates a regression line.

Finally, we analyzed the relationship between Lamin A or Emerin expression and nuclear morphological parameters using the CAIA data. We found that Lamin A expression was negatively correlated with the nuclear area (R = −0.376540) and perimeter (R = −0.332938) (Figures 6B,C). However, Emerin expression showed no correlation with the nuclear area (R = 0.104738) and nuclear perimeter (R = 0.086683) (Figures 6D,E). Thus, these data indicated that nuclear size enlargement in IPMC would be caused by decreased Lamin A expression, but not Emerin.

## Discussion

In this study, 30 (71.4%) specimens of IPMA and 12 (28.6%) specimens of IPMC were included (total of 42 IPMN specimens). The histological subtypes in this study included 26 (62.0%) gastric, 8 (19.0%) pancreatobiliary, 6 (14.3%) intestinal, and 2 (4.8%) oncocytic. In descending order, Andrejevic-Blant et al. [1] ordered the frequencies of IPMN subtypes as intestinal (54%), gastric (26%), oncocytic (13%), and pancreatobiliary (7%), whereas Mohri [21] reported that the gastric and intestinal subtypes were equally common (44% each), followed by the oncocytic (8%) and pancreatobiliary (4%) subtypes. In a Japanese study by Saito et al. [5], the gastric subtype was found the most common (59.3%), followed by the intestinal (25.6%), pancreatobiliary (12.8%), and oncocytic (2.3%) subtypes. Thus, the frequencies of IPMN subtypes observed in the present study are similar to those of Saito et al.

Using Feulgen-positive regions to analyze nuclear morphology, the present study found that the nuclear area and perimeter were significantly larger in IPMC than in IPMA. Since no reports about nuclear morphological analysis in IPMA and IPMC have been retrieved, we have had to compare our results with those of studies on other organs. In a study on thyroid follicular adenoma and follicular carcinoma, no significant differences were observed in the nuclear area and perimeter between the two neoplasm types [22]. By contrast, the nuclear area in colorectal carcinomas was larger than that in adenomas [23,24]. In addition, a study of lung lesions showed that the nuclear area tends to increase in the order of cellular atypia: low-grade atypical adenomatous hyperplasia (AAH), high-grade AAH, early bronchioloalveolar carcinoma (BAC), and obvious BAC [25].

A report that used the Gene Expression Profiling Interactive Analysis found that Lamin A expression in bladder urothelial carcinoma, kidney chromophobe carcinoma, acute myeloid leukemia, ovarian serous cystadenocarcinoma, pheochromocytoma and paraganglioma, prostate adenocarcinoma, testicular germ cell tumors, and uterine corpus endometrial carcinoma was lower than that in the normal tissue [12]. In the present study, the Lamin A-positive ratio in IPMC was found to be lower than that in IPMA. However, Lamin A expression in IPMA was mostly higher than that of the normal pancreatic duct. That is, Lamin A expression is increased in IPMA and decreased in IPMC but mostly not abolished in the IPMC. Regarding the relationship between Emerin expression and nuclear morphology, Liddane et al. reported that higher Emerin expression enlarged the nuclear size [26], whereas Lammerding et al. reported that Emerin suppression enlarged the nuclear size [11]. In the present study, we found that Emerin expression did not affect the nuclear size alteration. We also indicated that decreased Lamin A expression occurred during the malignant transformation of IPMA to IPMC, whereas Emerin expression would not significantly change during the malignant transformation of IPMA to IPMC.

Since we could not conduct a comparison between Lamin A expression and lymph node metastasis because only one case of IPMC had lymph node metastasis, further studies involving a larger number of specimens is necessary in the future. With regard the effect of Lamin A inhibition on tumor growth characteristics, Harada et al. [10] utilized a human A549 xenograft animal model to demonstrate that Lamin A knockdown cells grew more rapidly and tumor size increased compared with wild-type or scrambled siRNA-treated control A549 cells. The present and previous findings affirm that Lamin A expression not only promotes cancer metastasis but also supports subsequent tumor growth. Because no distant metastasis-positive cases were included in our study, we were not able to examine the association between Lamin A expression and distant metastasis. This aspect also needs further investigation involving larger datasets.

## Conclusion

Our results suggest that decreasing the Lamin A-positive ratio would induce nuclear enlargement and promote adenomas to carcinomas. Furthermore, Lamin A expression can be a useful biomarker for distinguishing between IPMC and IPMA. This is the first report on decreased Lamin A expression in IPMC cases.

## Data Availability

The datasets used and/or analyzed during the current study are available from the corresponding author on reasonable request.

## Supplementary material

SUPPLEMENTARY FIGURE S1 Representative examples of Feulgen-positive regions detected by computer-assisted image analysis. **(A)** Individual areas containing multiple nuclei. **(B)** Detected areas containing nonnuclear areas, although a single nucleus was detected. **(A,B)** Were excluded from this study. **(C)** Detected area containing a single nucleus. Such cases as **(C)** were used for nuclear morphological analysis.

SUPPLEMENTARY FIGURE S2 Comparison of Emerin expression in IPMN through manual evaluation and CAIA. **(A)** Contingency table of Emerin expression level evaluation by two pathologists between IPMA and IPMC. **(B,C)** Comparison of Emerin expression between IPMA and IPMC. **(B)** Box plots of Emerin expression in the total IPMA (left) and total IPMC (right). **(C)** Box plots of Emerin expression in the nongastric-type IPMA (left) and IPMC (right). The horizontal line within the boxes indicates the median and interquartile range. Error bars indicate maximum and minimum values. The mean was evaluated using Welch’s test, and *p* values were calculated using χ^2^ test.

SUPPLEMENTARY FIGURE S3 Scatterplot of Lamin A- and Emerin-positive ratios for all IPMN cases. The black square indicates gastric-type IPMA; white square, nongastric-type IPMA; black circle, gastric-type IPMC; and white circle, nongastric-type IPMC.
