# Supplementary material for: Negative correlation between the nuclear size and nuclear Lamina component Lamin A in intraductal papillary mucinous neoplasms of the pancreas
Source: Pathol Oncol Res. 2022 Dec 6;28:1610684. doi: 10.3389/pore.2022.1610684 (PMC9764245; doi:10.3389/pore.2022.1610684)
Supplement: Supplementary file 1 [file Table1.docx]

Supplemental Table 1. Summary of tumor subtypes and number of specimens

| Tumor subtype | IPMA | IPMC | Total |
| --- | --- | --- | --- |
| Gastric | 25 | 1 | 26 |
| Pancreatobiliary | 2 | 6 | 8 |
| Intestinal | 3 | 3 | 6 |
| Oncocytic | 0 | 2 | 2 |
| Total | 30 | 12 | 42 |

IPMA, intraductal papillary mucinous adenoma; IPMC, intraductal papillary

mucinous carcinoma.
