# Supplementary figures and images for: Negative correlation between the nuclear size and nuclear Lamina component Lamin A in intraductal papillary mucinous neoplasms of the pancreas
Source: Pathol Oncol Res. 2022 Dec 6;28:1610684. doi: 10.3389/pore.2022.1610684 (PMC9764245; doi:10.3389/pore.2022.1610684)

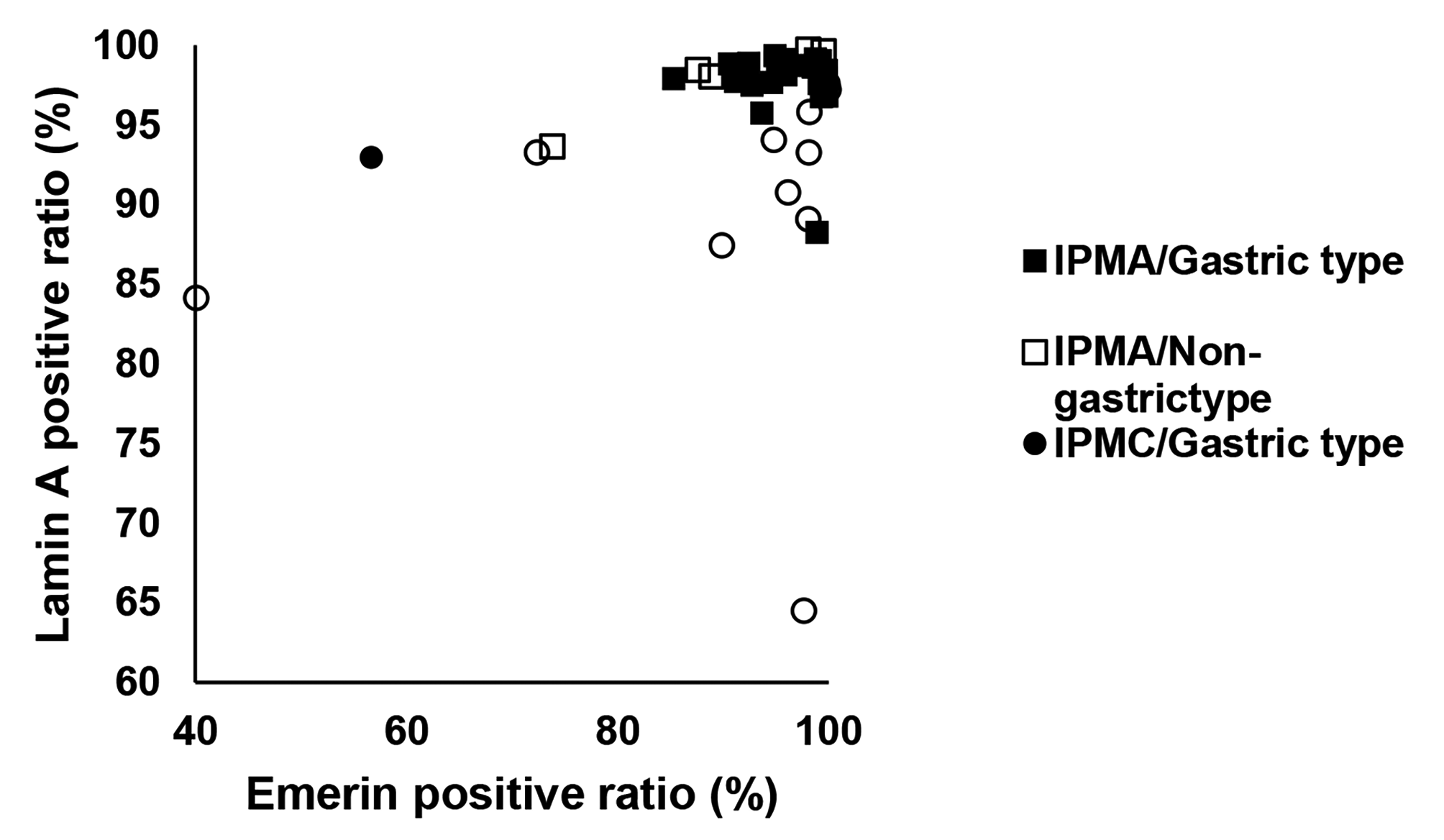

Supplement: Supplementary file 2 [file Image3.TIF]

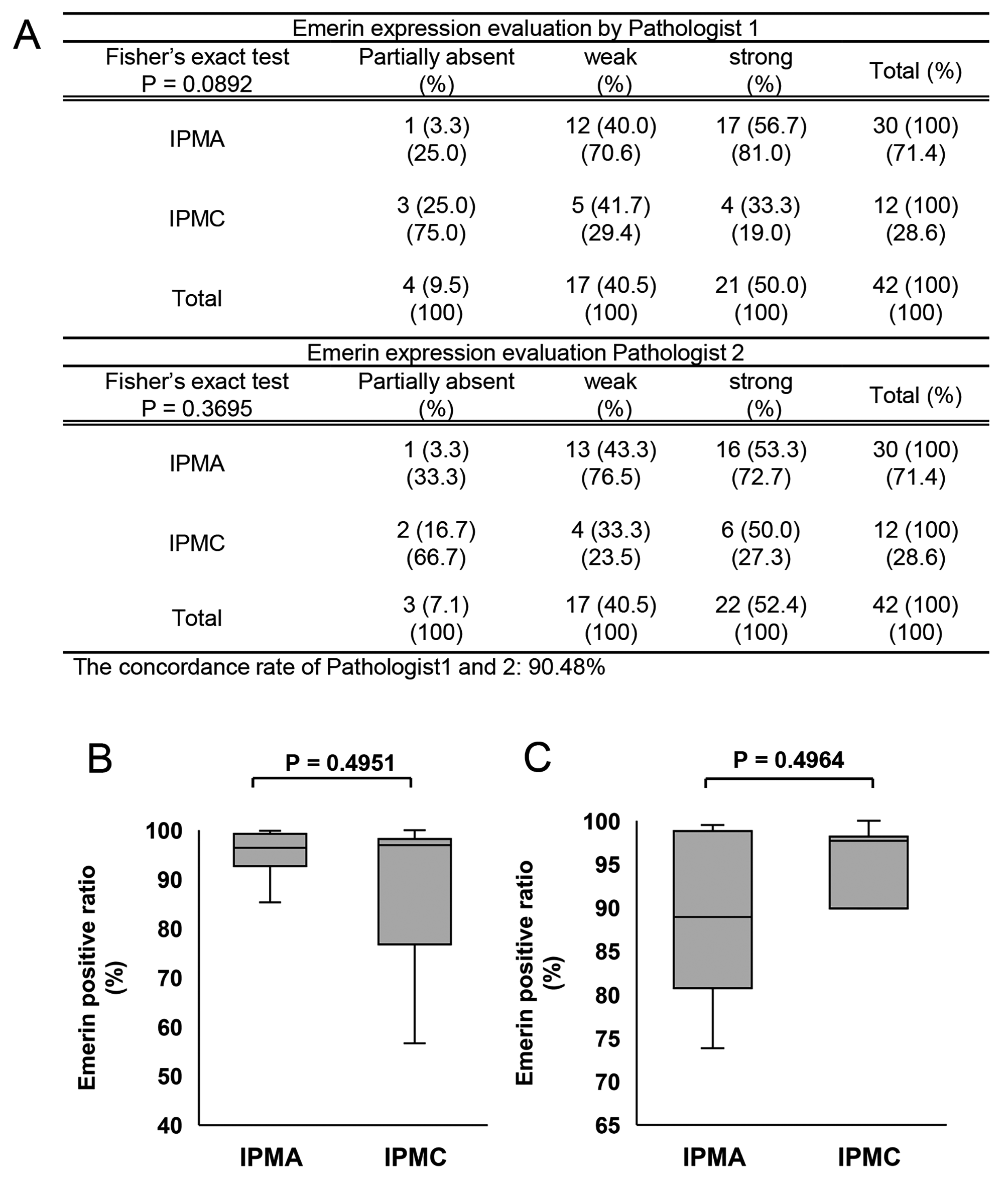

Supplement: Supplementary file 3 [file Image2.TIF]

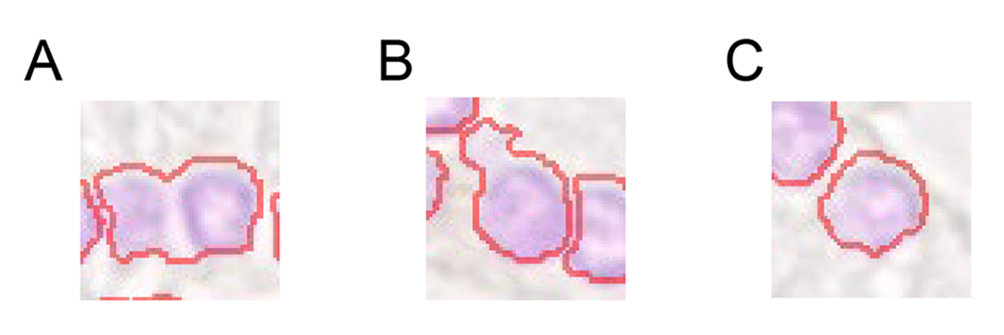

Supplement: Supplementary file 4 [file Image1.TIF]
