# Supplementary material for: Negative correlation between the nuclear size and nuclear Lamina component Lamin A in intraductal papillary mucinous neoplasms of the pancreas
Source: Pathol Oncol Res. 2022 Dec 6;28:1610684. doi: 10.3389/pore.2022.1610684 (PMC9764245; doi:10.3389/pore.2022.1610684)
Supplement: Supplementary file 5 [file Table2.docx]

Supplemental Table 2. Summary of tumor subtypes and number of specimens

|  | Gastric type | Non-gastric type | Total |
| --- | --- | --- | --- |
| IPMA | 25 (83.3%)  (96.1%) | 5 (16.7%)  (31.3%) | 30  (71.4%) |
| IPMC | 1 (8.3%)  (3.8%) | 11 (91.7%)  (68.8%) | 12  (28.6%) |
| Total | 26 (61.9%) | 16 (38.1%) | 42 |

IPMA, intraductal papillary mucinous adenoma; IPMC, intraductal papillary

mucinous carcinoma.
